# Influential Factors in the Efficacy and Safety of Hemoporfin‐Mediated Photodynamic Therapy for Facial Port‐Wine Stains

**DOI:** 10.1111/jocd.70153

**Published:** 2025-04-01

**Authors:** Ruixuan You, Yixin Tan, Xueqin Zhang, Lu Li, Ting Zeng, Yixin Zhou, Yaqian Shi, Yongqi Shao, Hongxia Yan, Rong Xiao, Xiangning Qiu

**Affiliations:** ^1^ Department of Dermatology The Second Xiangya Hospital of Central South University Changsha China; ^2^ Clinical Medical Research Center of Major Skin Diseases and Skin Health of Hunan Province The Second Xiangya Hospital of Central South University Changsha China; ^3^ Clinical Medical Research Center for Systemic Autoimmune Diseases in Hunan Province The Second Xiangya Hospital of Central South University Changsha China

**Keywords:** efficacy, hemoporfin, photodynamic therapy, port‐wine stain, safety

## Abstract

**Background:**

Hemoporfin‐mediated photodynamic therapy (HMME‐PDT) is a novel and promising treatment for port‐wine stains (PWS); however, the factors affecting the efficacy and safety are controversial.

**Aim:**

In this study, the relationship between influential factors and the efficacy and safety of HMME‐PDT for facial PWS was clarified.

**Methods:**

In this retrospective study, 155 patients suffering from facial PWS who underwent HMME‐PDT at 2–3 months interval were included. The efficacy was evaluated by the facial PWS area and severity index (FSASI) scoring system based on pre‐ and post‐treatment photographs of the lesion. The demographic characteristics, lesion patterns, treatment history, number of treatment sessions, and adverse events were also recorded.

**Results:**

After the first and second sessions of HMME‐PDT, 61.9% and 92.9% of patients responded, respectively. The lesion subtype, degree of lip involvement, and number of HMME‐PDT sessions were associated with the efficacy of HMME‐PDT (*p* = 0.014, *p* = 0.034, *p* < 0.001, and respectively). Logistic regression analysis confirmed that the patients with red‐type PWS (*p* = 0.019) and more HMME‐PDT sessions (≥ 2) (*p* < 0.001) showed an association with a better response to HMME‐PDT. The response rate was higher for lesions on the mandibular prominence area than that on the forehead area (*p* = 0.031). No photosensitivity or systemic adverse reactions were reported. Most adverse effects were well tolerated in all patients. Furthermore, the severity of crust was found positively associated with the efficacy (*p* < 0.001).

**Conclusion:**

HMME‐PDT is an effective and safe treatment approach for treating facial PWS. Patients who had red‐type, nonforehead area, and more treatment sessions showed an association with better efficacy.

## 1 Introduction

Port‐wine stain (PWS) is a common congenital and progressive malformation of capillaries or microvessels, with 0.3% to 0.5% incidence rates in newborns [1]. PWS often occurs on the face, neck, scalp, and any parts of the body during or shortly after birth. In its early stage, the skin lesions are usually flat and pink, with no protrusions above the skin surface [2]. With age, PWS gradually deepens to dark red or purple, and even becomes hypertrophic or nodular, and causes significant impairment to both physical and mental health [3]. Currently, pulsed dye laser (PDL) is regarded as the gold standard for treating PWS [4]; nonetheless, it remains limited in its effectiveness, as less than 10% of patients achieve complete clearance [5]. Furthermore, the recurrence, manifested as redarkening, is relatively high [6].

Hemoporfin, or hematoporphyrin monomethyl ether, is a novel second‐generation porphyrin‐based photosensitizer. Its several advantages include a strong photodynamic effect, low phototoxicity, rapid metabolism, and a brief light protection period [7]. Previous studies have demonstrated that hemoporfin‐mediated photodynamic therapy (HMME‐PDT) is remarkably effective and safe in treating PWS [8], even showing promising results in PDL‐resistant patients with PWS [9].

In the past clinical practice, dermatologists assessed the severity of facial PWS mainly relying on subjective judgment. Remarkably, our study was the first to use the facial PWS area and severity index (FSASI) scoring system for quantitative and comprehensive evaluation of the severity of facial PWS based on area, color, and thickness [10], thus improving the reliability and validity of our results.

Therefore, this study aimed to evaluate the factors influencing the efficacy and safety of HMME‐PDT in patients with facial PWS through the FSASI scoring system.

## 2 Materials and Methods

### 2.1 Study Design

Patients with clinically confirmed facial PWS who had undergone HMME‐PDT between March 2017 and October 2022 were enrolled in this study. The exclusion criteria were patients: (1) who used photosensitive drugs within 2 weeks prior to the treatment, (2) having skin photosensitivity, porphyria, or allergy to related drugs and ingredients, (3) suffering from other vascular syndromes, (4) unable to comply with follow‐up after HMME‐PDT treatment, or (5) who did not have clear photographs available for efficacy evaluation. All participating patients were informed about the treatment protocol, and they provided their consent by signing the informed consent forms. The following patient data were acquired: demographic characteristics, lesion patterns, treatment history, number of treatment sessions, adverse effects, and lesion photographs taken pre‐ and post‐HMME‐PDT treatment. The study protocol was approved by the Ethics Committee of the Second Xiangya Hospital of Central South University (LYF20240248).

### 2.2 Treatment Protocol

All patients were treated in the absence of anesthesia and sedation. Before treatment, the surrounding normal skin was covered fully, and the target area was exposed and marked. Then, the photosensitizer hemoporfin (Shanghai Fudan Zhangjiang Bio‐Pharmaceutical Co Ltd., Shanghai, China) was gradually injected intravenously at a dose of 5 mg/kg for 20 min in adults and 5 min in children. After 8 and 3 min of injection in adults and children, the target PWS lesions were exposed to 532 nm LED green light (Wuhan YaGe Photoelectric Technology Co. Ltd., China) for 15–20 min under the irradiation power density of 80–100 mw/cm^2^. Following each treatment, patients were instructed to avoid exposure to direct, strong light for 2 weeks and to intermittently apply ice packs to the skin lesions within 1 week. If repeated treatments were required, the treatment interval between each was generally 2–3 months. An optical camera was used to take photographs of the patient's marked skin lesions for documentation and evaluation before the initial treatment, at the end of the treatment cycle, and again 2–3 months later.

### 2.3 Evaluation Protocols

Three experienced dermatologists independently evaluated the clinical efficacy of the treatment based on photographs of lesions taken pre‐ and post‐treatment, according to the FSASI scoring system. The FSASI score for each patient was shown in Sheet S1. The degree of improvement = (FSASI score before treatment—FSASI score after treatment)/FSASI score before treatment. The criteria for evaluation were standardized as follows: poor (< 25% improvement), fair (≥ 25% and < 50% improvement), good (≥ 50% and < 75% improvement), and excellent response (≥ 75% improvement). A patient with improvement ≥ 25% was defined to be a responder. In cases where three dermatologists had disagreements, the evaluation was reassessed until consensus was achieved, ensuring that the findings were accurate and consistent.

According to the vascular classification guidelines proposed by Waelchli et al. facial lesions were categorized into forehead area, maxillary prominence area, and mandibular prominence area based on the embryonic origin of blood vessels [11].

Local adverse effects occurring during and after HMME‐PDT treatment, such as itch, burning, pain, edema, blister, crust, hyperpigmentation, hypopigmentation, infection, and scar, with the degrees, including none, mild, moderate, and severe, were recorded in detail. The patients self‐reported the severity of itch, burning, and pain, while the dermatologists evaluated the severity of other adverse effects. This comprehensive approach ensured accurate documentation and assessment of the potential side effects associated with HMME‐PDT.

### 2.4 Statistical Analysis

Demographic characteristics were descriptively summarized, and categorical variables were presented in terms of numbers with percentages. The Kruskal–Wallis test was used to compare the class of efficacy across the groups. The chi‐square test or Fisher's exact test was applied to explore associations between influential factors and the response rate. Independent factors affecting the treatment efficacy were further determined by multivariate ordinal logistic regression analysis. The analysis results were expressed as odds ratio (OR) values and the corresponding 95% confidence intervals (CI). To assess the linear correlation in adverse effects across the groups, the nonparametric Spearman correlation test was applied. The statistical analyzes were conducted in SPSS Statistics version 27.0. All *p*‐values were two‐sided, and *p* < 0.05 was considered statistically significant.

## 3 Results

### 3.1 Demographic Characteristics

In this study, a total of 155 cases with facial PWS were enrolled. The patient cohort comprised 61 (39.4%) males and 94 (60.6%) females, in ages ranging from 1 to 54 years. In terms of the color of their PWS, there were 28 (18.1%) patients with red‐type, 108 (69.7%) with purple‐type, and 19 (12.3%) with hypertrophic‐type PWS. Among the included patients, lip involvement without hypertrophy was observed in 94 (60.6%) cases, lip involvement without hypertrophy was observed in 49 (31.6%) cases, and those with hypertrophy were observed in 12 (7.7%) cases. Additionally, 52 (33.5%) patients had a prior treatment history of PDL, cryotherapy, and isotope therapy. The baseline characteristics of the patients are summarized in Table 1, which provides a comprehensive overview of the study population.

**TABLE 1:** Summary of patient characteristics and comparison of the efficacy according to different factors after the first session of HMME‐PDT.

Factors	*N*	Class of efficacy (%)			*Z*	*p*
		Poor	Fair	Good		
Total	155	59 (38.1)	67 (43.2)	29 (18.7)		
Age					3.094	0.542
1–3	11 (7.1)	4 (36.4)	6 (54.5)	1 (9.1)		
3–6	39 (25.2)	13 (33.3)	15 (38.5)	11 (28.2)		
6–14	31 (20.0)	11 (35.5)	14 (45.2)	6 (19.4)		
14–25	41 (26.5)	19 (46.3)	17 (41.5)	5 (12.2)		
> 25	33 (21.3)	12 (36.4)	15 (45.5)	6 (18.2)		
Sex					1.642	0.200
Female	94 (60.6)	34 (36.2)	38 (40.4)	22 (23.4)		
Male	61 (39.4)	25 (41.0)	29 (47.5)	7 (11.5)		
Subtype					8.497	0.014
Red	28 (18.1)	11 (39.3)	7 (25.0)	10 (35.7)		
Purple	108 (69.7)	35 (32.4)	55 (50.9)	18 (16.7)		
Hypertrophic	19 (12.3)	13 (68.4)	5 (26.3)	1 (5.3)		
Lip involvement					6.779	0.034
Hypertrophy	12 (7.7)	8 (66.7)	3 (25.0)	1 (8.3)		
Without hypertrophy	49 (31.6)	21 (42.9)	22 (44.9)	6 (12.2)		
None	94 (60.6)	30 (31.9)	42 (44.7)	22 (23.4)		
Other treatment prior to HMME‐PDT					0.039	0.844
No	103 (66.5)	39 (37.9)	44 (42.7)	20 (19.4)		
Yes	52 (33.5)	20 (38.5)	23 (44.2)	9 (17.3)		

Abbreviation: HMME‐PDT, hemoporfin‐mediated photodynamic therapy.

### 3.2 Effectiveness

**FIGURE 1:**
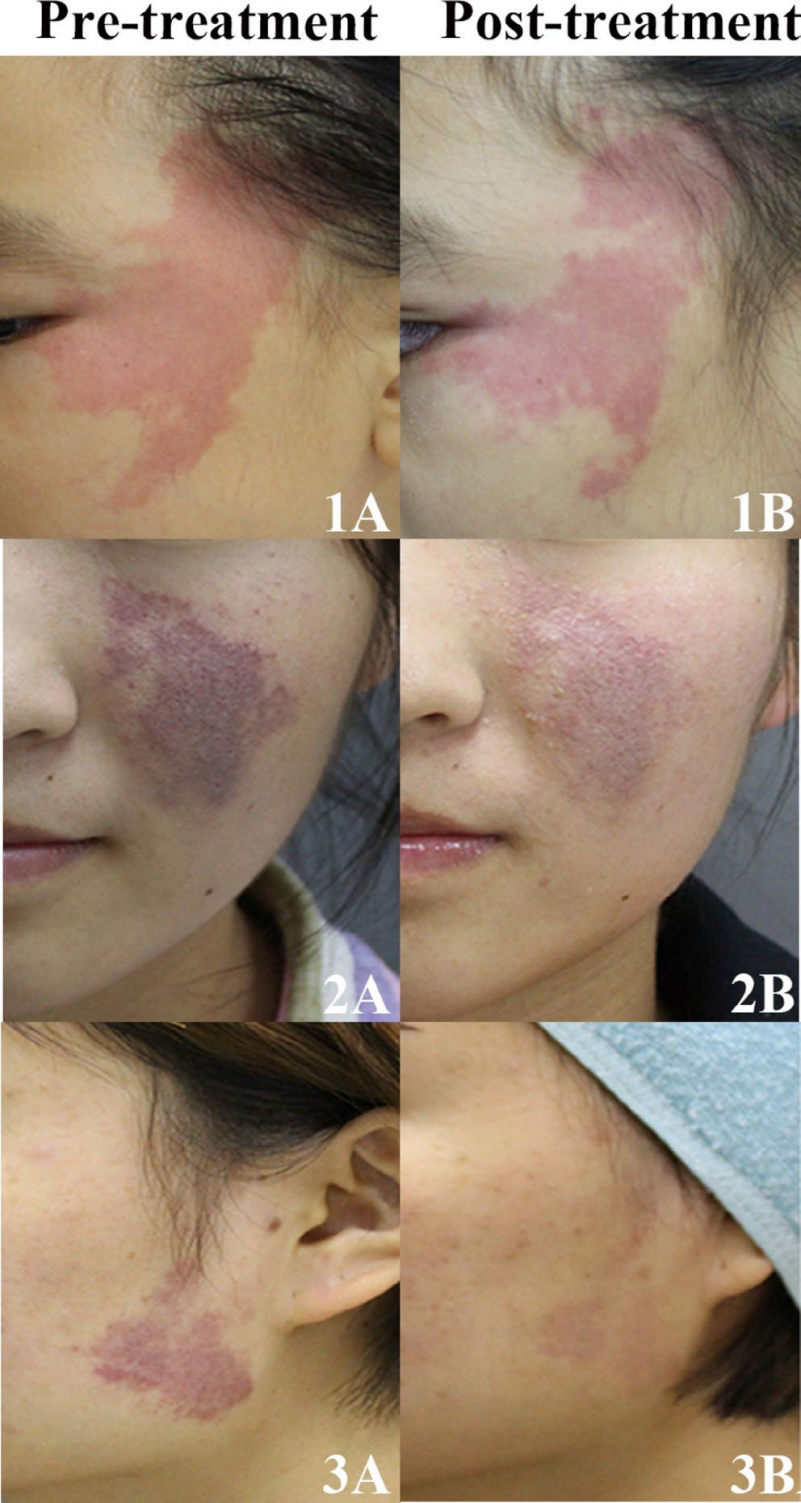
Representative photographs of patients before and after the first session of HMME‐PDT. A facial PWS showing < 25% improvement (poor): 1A, 1B. A facial PWS showing ≥ 25% and < 50% improvement (fair): 2A, 2B. A facial PWS showing ≥ 50% and < 75% improvement (good): 3A, 3B.

After the first session of HMME‐PDT, 61.9% of patients responded. The patient response was categorized as follows: 29 (18.7%) patients exhibited a good response, 67 (43.2%) showed a fair response, and 59 (38.1%) demonstrated a poor response. During the follow‐up period, 99 patients were evaluated for efficacy after two sessions of HMME‐PDT (Table S1). The response rate after two sessions of treatment was 92.9%, which was significantly higher than that after the first session (*p* < 0.001). Representative photographs of patients with PWS before and after first session of treatment are presented in Figure 1.

According to univariate analysis, the lesion subtype and the degree of lip involvement significantly impacted the efficacy of the first HMME‐PDT (*p* = 0.014 and *p* = 0.034). However, there was no significant difference in the response to HMME‐PDT treatment among patients of different ages, sexes, or previous treatment histories following the first session of HMME‐PDT (Table 1).

Results of multivariate logistic regression analysis in Table 2 revealed that the hypertrophic‐type PWS responded worse to the treatment than the red‐type PWS (OR: 0.231, 95% CI: 0.068–0.783, *p* = 0.019), while the response in the purple‐type was comparable to that of the red‐type (OR: 0.860, 95% CI: 0.387–1.908, *p* = 0.710). Moreover, the treatment outcomes among patients stratified by the degree of lip involvement did not differ at statistical significance levels (*p* > 0.05).

**TABLE 2:** The multivariate logistic regression analysis on factors affecting the efficacy of HMME‐PDT.

Factors	*β*	SE	Wald	*p*	OR	95% CI
Subtype						
Red	Ref.	—	—	—	—	—
Purple	−0.151	0.407	0.138	0.710	0.860	0.387–1.908
Hypertrophic	−1.466	0.624	5.530	0.019	0.231	0.068–0.783
Lip involvement						
None	Ref.	—	—	—	—	—
Without hypertrophy	−0.577	0.345	2.794	0.095	0.562	0.286–1.104
Hypertrophy	−1.119	0.664	2.842	0.092	0.327	0.089–1.200
Number of HMME‐PDT sessions						
1	Ref.	—	—	—	—	—
2	1.838	0.386	22.673	< 0.001	6.284	2.948–13.383
≥ 3	2.435	0.440	30.569	< 0.001	11.416	4.816–27.058

Abbreviation: HMME‐PDT, hemoporfin‐mediated photodynamic therapy.

Among the enrolled patients, 56 (36.1%) received one session of HMME‐PDT, 58 (37.4%) received two sessions, and 41 (26.5%) received more than three sessions. Both univariate and multivariate analyzes indicated that the number of HMME‐PDT sessions was an independent influential factor for the efficacy (Table 2; Table S2). Compared to patients who underwent one session, those who underwent two or more than three sessions had a better response (*p* < 0.001).

Regarding the facial regions, 22 (18.0%) had PWS lesions predominantly on the forehead area, 61 (50.0%) had lesions exclusively on the maxillary prominence area, and 39 (32.0%) had lesions solely on the mandibular prominence area. The response rate in different facial regions was 59.1%, 80.3%, 87.2%, and respectively (Table S3). The response rate of the forehead area was worse than that of the mandibular prominence area (*p* = 0.031), and the response rate of the maxillary prominence area was similar to the forehead area(*p* = 0.113) and mandibular prominence area (*p* > 0.999). We further explored the factors affecting the response rate for each facial region individually in Table 3. The results showed that the response of the maxillary prominence area was related to the lesion subtype (*p* = 0.037). However, no significant difference was found among patients stratified by ages, sexes, or previous treatment histories in any facial region (*p* > 0.05).

**TABLE 3:** Comparison of the response rate of HMME‐PDT in different facial regions.

Facial region	*p*
Forehead area	
Age	0.806
Sex	0.666
Subtype	> 0.999
Other treatment prior to HMME‐PDT	0.655
Maxillary prominence area	
Age	0.882
Sex	0.940
Subtype	0.037
Other treatment prior to HMME‐PDT	0.698
Mandibular prominence area	
Age	0.430
Sex	> 0.999
Subtype	0.052
Other treatment prior to HMME‐PDT	0.631

Abbreviation: HMME‐PDT, hemoporfin‐mediated photodynamic therapy.

### 3.3 Adverse Effects

At the time of HMME‐PDT treatment, most patients experienced various adverse effects, including itch (51.0%), burning (99.4%), pain (97.4%), and edema (100.0%). Post‐HMME‐PDT treatment, adverse effects included the development of blister (14.2%), crust (55.5%), hyperpigmentation (63.2%), hypopigmentation (4.5%), infection (2.6%), and scar (3.9%) (Table S4). None of the patients reported any serious adverse effects during or after the treatment.

After the first HMME‐PDT treatment, burning and pain were positively correlated with patient age and lesion subtype (*p* < 0.001), indicating a tendency for worse burning and pain in older patients or those with a more severe lesion subtype. Additionally, our assessment of the HMME‐PDT treatment response and adverse effects revealed a positive correlation between crust and treatment outcome, indicating a potentially higher chance of achieving a better response in patients with a more severe crust (*p* < 0.001) (Table 4).

**TABLE 4:** The association between patient characteristics, efficacy, and severity of adverse effects of HMME‐PDT.

		Itch	Burning	Pain	Edema	Blister	Crust	Hyperpigmentation	Hypopigmentation	Infection	Scar
Age	γ	−0.189*	0.438**	0.525**	0.263**	0.253**	−0.093	−0.040	−0.039	0.004	−0.145
	P	0.018	< 0.001	< 0.001	0.001	0.001	0.248	0.618	0.628	0.958	0.072
Sex	γ	0.017	0.112	0.149	0.138	0.029	0.107	0.046	0.048	0.048	−0.044
	P	0.835	0.164	0.063	0.088	0.722	0.186	0.571	0.553	0.550	0.589
Subtype	γ	−0.067	0.374**	0.381**	0.299**	−0.003	−0.103	−0.066	−0.031	−0.058	−0.164*
	P	0.406	< 0.001	< 0.001	< 0.001	0.974	0.201	0.417	0.699	0.476	0.042
Other treatment prior to HMME‐PDT	γ	−0.084	−0.080	−0.077	−0.239**	−0.057	0.026	0.115	0.240**	0.141	0.141
	P	0.298	0.322	0.340	0.003	0.481	0.747	0.153	0.003	0.080	0.081
Lip involvement	γ	−0.014	0.221**	0.179*	0.156	0.002	−0.043	−0.006	−0.032	−0.129	−0.097
	P	0.862	0.006	0.026	0.052	0.979	0.599	0.938	0.691	0.111	0.232
Class of efficacy	γ	0.104	−0.133	−0.104	−0.178*	0.079	0.319**	−0.037	0.020	0.107	0.010
	P	0.197	0.099	0.197	0.027	0.330	< 0.001	0.651	0.803	0.185	0.905

*Note:* Data were digitized as following, Severity of adverse effects –“0” none, “1” mild, “2” moderate, and “3” severe; Age‐“0” 1–3, “1” 3–6, “2” 6–14, “3” 14–25, and “4” > 25; Sex‐“0” male and “1” female; Subtype‐ “0” red, “1” purple, and “2” hypertrophic; Other treatment prior to HMME‐PDT‐ “0” no and “1” yes; Lip involvement – “0” none, “1” without hypertrophy, and “2” Hypertrophy; Class of response “1” poor, “2” fair, and “3”good. Spearman's rank order correlation coefficient γ (two‐tailed). *p < 0.05 and **p < 0.01.

Abbreviation: HMME‐PDT, hemoporfin‐mediated photodynamic therapy.

## 4 Discussion

PWS typically affects the face, causing cosmetic anxieties, significant psychological pressure, and compromised quality of life. HMME‐PDT is a novel therapy, now increasingly being used in the treatment of PWS due to its remarkable efficacy and good tolerance. Although the factors affecting its efficacy have become a research hotspot, the results of different studies vary greatly. This may be due to the lack of reliable criteria for determining the degree of efficacy of HMME‐PDT. Therefore, in this study, we applied the FSASI scoring system to reliably explore certain factors affecting the efficacy and safety of HMME‐PDT treatment in treating facial PWS. We observed that the lesion subtype, treatment session, and facial region were the primary factors affecting the therapeutic effect.

Generally, the response of younger patients to HMME‐PDT was better. However, upon further analysis, we found that age alone was not a significant factor influencing the efficacy of HMME‐PDT. Likewise, previous studies also revealed no statistically significant difference in efficacy among children, adolescents, and adults [12]. Nevertheless, with the increase of age, PWS lesions may tend to grow larger, darken, and become thicker, potentially seriously impacting social activities. According to our unpublished data, for patients older than 14 years old, the efficacy of HMME‐PDT showed an association with the lesion subtype (*p* = 0.035). This was attributed to the fact that the older the age, the higher the proportion of patients whose lesions developed hypertrophic‐type PWS, and the treatment response of hypertrophic‐type PWS was significantly worse than that of red or purple‐type PWS. Therefore, early detection, diagnosis, and treatment remain crucial. Additionally, there were no significant differences in the efficacy between different sexes after one session of HMME‐PDT treatment.

This study indicated whether or not the patients with previous treatment history will not affect the efficacy of HMME‐PDT, a finding that aligns with previous research [13]. Considering both efficacy and safety, HMME‐PDT is at least as effective as PDL, and in certain instances, it surpasses the latter [5]. Some studies also showed the efficacy of HMME‐PDT in treating facial PWS patients who are resistant to PDL [14]. Therefore, the efficacy of HMME‐PDT may not be affected by treatment history.

Both univariate and multivariate analyzes showed that the lesion subtype significantly affected the efficacy of HMME‐PDT, with red‐type PWS demonstrating a significantly better response than hypertrophic‐type PWS, and the response being comparable to that of purple‐type PWS. Even in different facial regions, the lesion subtype was still the main factor influencing the response rate. This result was consistent with previous research findings [12, 15]. This difference in efficacy among the lesion subtype could be attributed to the histological differences observed in the classifications of vascular patterns of the three types of PWS. Hypertrophic skin lesions have thicker vessels with relatively larger diameters and deeper locations, which can hinder the penetration of PDT light [16]. Additionally, different subtypes of PWS may exhibit vessels with distinct configurations, arrangements, connective tissue density, and the distributions of cutaneous appendages [17]. All the above parameters can potentially influence light energy or penetrability, consequently weakening the intensity of photochemical reactions. Therefore, we advise a comprehensive examination of the patient before the treatment, combined with dermoscopy and the VISIA system, to analyze the vascular structure and evaluate the expected treatment efficacy.

In our cohort, 94 patients had no lip involvement, 49 had lip involvement, and 12 presented with lip hypertrophy. The patients without lip involvement achieved the highest treatment effectiveness compared to those with lip involvement or hypertrophy. According to Ma et al., GNAQ mutations, highly enriched in the lip mucosa, may cause soft‐tissue hypertrophy and port‐wine macrocheilia [18]. Mechanistically, somatic mutations in GNAQ lead to significant activation of the mitogen‐activated protein kinase (MAPK)/extracellular signal‐regulated kinase (ERK) pathway, increased cell proliferation, apoptotic inhibition, and malformed vessels [19]. However, the mechanism of HMME‐PDT therapy mainly involves induced endothelial cell apoptosis and autophagy through oxidative stress [20]. This suggests that due to their underlying physiological characteristics, patients with a genetic predisposition towards lip hypertrophy may respond poorly to HMME‐PDT treatment. Currently, clinicians are exploring the use of a long‐pulsed 1064 nm laser for the treatment of hypertrophic‐type PWS, leveraging its deeper penetration capabilities, and have achieved promising results [21, 22].

Different from the traditional distribution of the trigeminal nerve, we followed the facial embryonic vascular system proposed by Waelchli R et al. to divide facial PWS into the forehead area, maxillary prominence area, and mandibular prominence area [11]. Our analyzes revealed that the response rate in the forehead area was lower than that in the maxillary prominence area or mandibular prominence area. Consistent with this phenomenon, one recent study reported that compared to other locations, satisfactory clearance was particularly challenging in the frontal and nasal skin lesions [15]. More importantly, patients with forehead area involvement should undergo an ophthalmologic examination and a brain MRI as early as possible to screen for Sturge–Weber syndrome (SWS). The possible explanation was that the developing vasculature of the forehead area was formed by the migration of neural crest cells from the forebrain and anterior midbrain, whereas the maxillary and mandibular prominences were formed by the migration of neural crest cells from the posterior mesencephalon and rhombencephalon [23]. It was precisely because of the variation in the embryological origin of the face that caused differences in the response rate. Furthermore, the cerebral cortex and eyes also developed from the forebrain, so patients with forehead area involvement were prone to confer a higher risk of SWS. Of course, the generalization of our findings on the correlation between efficacy and facial regions needs to be investigated further to discover the physiological, morphological, and anatomical traits of vessels in specific regions.

Previous studies, including ours, have yielded that the efficacy of HMME‐PDT was currently recognized to be positively related to the number of sessions [13, 15], called the cumulative effect. However, multiple treatments over a long period test the patient's compliance. In our study, the lesion subtype and facial region were notable factors concerning efficacy. Therefore, in clinical practice, dermatologists must emphasize the importance of perseverance in treatment, especially in encouraging patients with hypertrophic‐type PWS or lesions in the forehead area to attain improved long‐term clearance outcomes.

Our study found that most adverse reactions due to HMME‐PDT are temporary and self‐limited, with edema and pain being the most common adverse effects. In addition, with the increase in age and the severity of lesion subtype, patients experienced a higher incidence of pronounced burning and pain. Therefore, it is highly important to take proactive measures to mitigate these symptoms. Generally, chill wind is applied to improve pain tolerance during treatment, and oral corticosteroids are prescribed to relieve edema after treatment. Importantly, crust has been identified as a positive factor influencing treatment efficiency, consistent with previous studies [24]. The possible explanation is that crust represents a better response of vascular endothelial cells to HMME‐PDT [25]. Therefore, even if crust occurs, patients should not be too anxious and should also focus on preventing the risk of scarring due to crust, and strive for a balance between the two to achieve optimal treatment outcomes while preserving aesthetic appearance.

This study had a few limitations that need to be acknowledged. First, this was a retrospective design. The assessment of efficacy primarily relies on previously taken photographs of the lesion, and the inclusion of patients would introduce selection bias (limited sample size) or information bias, which may affect the reliability of the findings. Second, the short follow‐up time makes it difficult to address the long‐term efficacy and safety of HMME‐PDT. Third, our findings are primarily based on data from the Chinese population. Given the potential difference in gene mutation spectrum or vascular patterns between the Chinese population and other ethnic groups, caution should be exercised when applying HMME‐PDT in other populations. Consequently, it is imperative to conduct large‐scale, long‐term follow‐up prospective studies in various regions and among diverse populations to validate the efficacy and safety of HMME‐PDT.

## 5 Conclusion

The current study provides evidence that HMME‐PDT is effective as well tolerated in patients with facial PWS. Patients who had red‐type, nonforehead areas and more treatment sessions had a better response to the HMME‐PDT treatment. In addition, crust has a mild correlation with a better efficiency in this study. Investigations should be conducted to delve deeper into the pathophysiologic mechanisms involved in the crosstalk between lesion characteristics and efficacy.

## Author Contributions

**Ruixuan You:** writing – original draft, methodology, formal analysis. **Yixin Tan:** writing – original draft, methodology, formal analysis. **Xueqin Zhang:** writing – review and editing, investigation, resources, data curation. **Lu Li:** writing – review and editing, investigation, resources, data curation. **Ting Zeng:** writing – review and editing, investigation, resources, data curation. **Yixin Zhou:** writing – review and editing, investigation, resources, data curation. **Yaqian Shi:** writing – review and editing, investigation, resources, data curation. **Yongqi Shao:** writing – review and editing, investigation, resources, data curation. **Hongxia Yan:** writing – review and editing, investigation, resources, data curation. **Rong Xiao:** conceptualization, supervision, project administration. **Xiangning Qiu:** conceptualization, supervision, project administration. All authors have read and approved the final manuscript.

## Ethics Statement

This study was approved by the Ethics Committee of the Second Xiangya Hospital of Central South University (LYF20240248).

## Consent

Patients signed informed consent regarding the publishing their data and photographs.

## Conflicts of Interest

The authors declare no conflicts of interest.

## Supporting information


Tables S1–S4.

Data Sheet 1.

## Data Availability

All data generated or analyzed during this study are included in this published article. Additional data related to this study can be directed to the corresponding author.
