# Supplementary material for: Influential Factors in the Efficacy and Safety of Hemoporfin‐Mediated Photodynamic Therapy for Facial Port‐Wine Stains
Source: J Cosmet Dermatol. 2025 Apr 1;24(4):e70153. doi: 10.1111/jocd.70153 (PMC11959330; doi:10.1111/jocd.70153)
Supplement: Supplementary file 1 — Tables S1–S4. [file JOCD-24-e70153-s002.docx]

| Factors | N | Class of efficacy (%) | | | | Z | *p* |
| --- | --- | --- | --- | --- | --- | --- | --- |
|  |  | Poor | Fair | Good | Excellent |  |  |
| Total | 99 | 7(7.1) | 40(40.4) | 45(45.4) | 7(7.1) |  |  |
| Age | | | | |  | 3.649 | 0.456 |
| 1−3 | 6(6.1) | 0(0.0) | 3(50.0) | 3(50.0) | 0(0.0) |  |  |
| 3−6 | 23(23.2) | 2(8.7) | 5(21.7) | 15(65.2) | 1(4.3) |  |  |
| 6−14 | 22(22.2) | 1(4.5) | 9(40.9) | 10(45.5) | 2(9.1) |  |  |
| 14−25 | 26(26.3) | 1(3.8) | 16(61.5) | 8(30.8) | 1(3.8) |  |  |
| >25 | 22(22.2) | 3(13.6) | 7(31.8) | 9(40.9) | 3(13.6) |  |  |
| Sex | | | | |  | 0.031 | 0.860 |
| Female | 64(64.6) | 3(4.7) | 28(43.8) | 28(43.8) | 5(7.8) |  |  |
| Male | 35(35.4) | 4(11.4) | 12(34.3) | 17(48.6) | 2(5.7) |  |  |
| Subtype | | | | |  | 18.366 | <0.001 |
| Red | 16(16.2) | 0(0.0) | 4(25.0) | 10(62.5) | 2(12.5) |  |  |
| Purple | 67(67.7) | 2(3.0) | 27(40.3) | 33(49.3) | 5(7.5) |  |  |
| Hypertrophic | 16(16.2) | 5(31.3) | 9(56.3) | 2(12.5) | 0(0.0) |  |  |
| Other treatment prior to HMME-PDT | | | | |  | 0.528 | 0.467 |
| No | 64(64.6) | 6(9.4) | 26(40.6) | 27(42.2) | 5(7.8) |  |  |
| Yes | 35(35.4) | 1(2.9) | 14(40.0) | 18(51.4) | 2(5.7) |  |  |
| Lip involvement | | | | |  | 2.269 | 0.322 |
| Hypertrophy | 10(10.1) | 2(20.0） | 5(50.0) | 2(20.0) | 1(10.0) |  |  |
| Without hypertrophy | 34(34.3) | 2(5.9) | 14(41.2) | 15(44.1) | 3(8.8) |  |  |
| None | 55(55.6) | 3(5.5) | 21(38.2) | 28(50.9) | 3(5.5) |  |  |

Supplemental Table 1 Comparison of the efficacy of HMME-PDT according to different factors after two sessions

Abbreviations: HMME-PDT, hemoporfin-mediated photodynamic therapy

| Factors | N | Class of efficacy (%) | | | | Z | *p* |
| --- | --- | --- | --- | --- | --- | --- | --- |
|  |  | Poor | Fair | Good | Excellent |  |  |
| Total | 155 | 12(7.7) | 49(31.6) | 71(45.8) | 23(14.8) |  |  |
| Number of HMME-PDT sessions | | | | |  | 36.912 | <0.001 |
| 1 | 56(36.1) | 9(16.1) | 29(51.8) | 18(32.1) | 0(0.0) |  |  |
| 2 | 58(37.4) | 1(1.7) | 15(25.9) | 31(53.4) | 11(19.0) |  |  |
| ≥3 | 41(26.5) | 2(4.9) | 5(12.2) | 22(53.7) | 12(29.3) |  |  |

Supplemental Table 2 Comparison of the efficacy according to the number of HMME-PDT sessions

Abbreviations: HMME-PDT, hemoporfin-mediated photodynamic therapy

| Factors | N | Class of efficacy (%) | | | Responder N (%) | χ^2^ | *p* |
| --- | --- | --- | --- | --- | --- | --- | --- |
|  |  | Poor | Fair | Good |  |  |  |
| Total | 122 | 26(21.3) | 67(54.9) | 29(23.8) | 96(78.7) |  |  |
| Facial regions | | | | |  | 6.813 | 0.033 |
| Forehead area | 22(18.0) | 9(40.9) | 8(36.4) | 5(22.7) | 13(59.1) |  |  |
| Maxillary prominence area | 61(50.0) | 12(19.7) | 34(55.7) | 15(24.6) | 49(80.3) |  |  |
| Mandibular prominence area | 39(32.0) | 5(12.8) | 25(64.1) | 9(23.1) | 34(87.2) |  |  |

Supplemental Table 3 Comparison of the efficacy of HMME-PDT according to the facial regions

Abbreviations: HMME-PDT, hemoporfin-mediated photodynamic therapy

Supplemental Table 4 Adverse effects of HMME-PDT after first session

| Adverse effects | N | Class of adverse effects (%) | | | |
| --- | --- | --- | --- | --- | --- |
|  |  | None | Mild | Moderate | Severe |
| Itch | 155 | 76(49.0) | 65(41.9) | 9(5.8) | 5(3.2) |
| Burning |  | 1(0.6) | 43(27.7) | 50(32.3) | 61(39.4) |
| Pain |  | 4(2.6) | 36(23.2) | 39(25.2) | 76(49.0) |
| Edema |  | 0(0.0) | 110(71.0) | 41(26.5) | 4(2.6) |
| Blister |  | 133(85.8) | 20(12.9) | 2(1.3) | 0(0.0) |
| Crust |  | 69(44.5) | 85(54.8) | 1(0.6) | 0(0.0) |
| Hyperpigmentation |  | 57(36.8) | 88(56.8) | 10(6.5) | 0(0.0) |
| Hypopigmentation |  | 148(95.5) | 7(4.5) | 0(0.0) | 0(0.0) |
| Infection |  | 151(97.4) | 3(1.9) | 1(0.6) | 0(0.0) |
| Scar |  | 149(96.1) | 6(3.9) | 0(0.0) | 0(0.0) |

Abbreviations: HMME-PDT, hemoporfin-mediated photodynamic therapy
